# Polymorphism of keratin 1 associates with systemic lupus erythematosus and systemic sclerosis in a south Chinese population

**DOI:** 10.1371/journal.pone.0186409

**Published:** 2017-10-13

**Authors:** Weiguang Luo, Bin Zhou, Qizhi Luo, Huilong Fang, Xiaoxia Zuo, Yizhou Zou

**Affiliations:** 1 Department of Immunology, Xiangya School of Medicine, Central South University, Changsha, Hunan, China; 2 Department of Rheumatology, Xiangya Hospital, Central South University, Changsha, Hunan, China; 3 Department of pathogenic Biology and Immunology, Xiangnan University, Chenzhou, Hunan, China; 4 The Cooperative Innovation Center of Engineering and new Products for Developmental Biology of Hunan Province, Changsha, Hunan, China; NIDCR/NIH, UNITED STATES

## Abstract

Both systemic lupus erythematosus (SLE) and systemic sclerosis (SSc) diseases are related to the genetic and environmental factors, causing damage to the skin. The mutations of keratin 1 gene (*KRT1*) were reported to associate with skin diseases. The single-nucleotide polymorphism (SNP, rs14024) and the indel polymorphism (cds-indel, rs267607656), consisting mostly of the common haplotypes and could be used for genotyping of *KRT1*. We used the PCR with sequence specific primers (PCR-SSP) to determine the genotype of *KRT1* in 164 SLE, 99 SSc patients, and 418 healthy controls. The results showed that the mutant with G at SNP rs14024 was associated with the high risk to SLE (p = 6.48×10^−5^) and SSc (p = 8.75×10^−5^), while the deletion allele at rs267607656 was associated with the low risk to SSc (p = 4.89×10^−4^) comparing to the normal controls. Haplogenotype, Del-/MU+ was associated with high susceptibility to SLE (OR = 1.87, p = 0.001) and SSc (OR = 2.29, p = 2.34×10^−4^). In contrast, the Haplogenotype Del+/MU- was associated with resistance to SLE (OR = 0.35, p = 6.24×10^−5^) and SSc (OR = 0.34, p = 0.001). This study demonstrates that the variations in *KRT1* and the specific polymorphism of *KRT1* in this Chinese Han population are associated with autoimmune diseases SLE and SSc. Typing *KRT1* might be helpful to identify SLE and SSc patients.

## Introduction

Systemic lupus erythematosus (SLE) is an autoimmune inflammatory disease with symptoms that can affect almost any organ [1]. The disease is caused by the deposition of antibody and immune complexes in blood vessels, leading to inflammation in the skin, joints, serosa, central nervous system, and/or kidney. Both genetic and environmental factors contribute to development of the pathology [2]. Systemic sclerosis (SSc) is a systemic connective tissue disease, also of autoimmune origin [3]. Patients suffer from excessive accumulation of extracellular matrix that results in progressive fibrotic replacement of normal tissue architecture [4], which affects the skin and internal organs. Recent genome-wide association studies (GWAS) and candidate gene studies have confirmed genetic associations of 18 loci with SLE susceptibility achieving genome-wide significance in Asian population [5–8]. Mayes MD et al have found that seven SNPs in the human leukocyte antigen (HLA) gene region and three non-HLA loci are associated with SSc diseases using the immunochip array [9].

These loci do not fully explain the genetic susceptibility to SLE and/or SSc, suggesting additional genetic factors yet to be discovered. Keratins are well known as intermediate filament forming proteins in epithelial cells [10]. Based on physicochemical properties on cells and tissues, keratins are distinguished as type I (KRT9–KRT28; KRT31–KRT38) and type II (KRT1–KRT8; KRT71–KRTT86) [11]. KRT1 and its heterodimer partner KRT10 are produced in the suprabasal cells of the epidermis[12] and are considered to be the earliest markers of terminal differentiation[13, 14]. KRT1 and KRT10 form the major filament cytoskeleton that provides mechanical support and involves in additional functions in epithelial cells [15]. KRT1, without KRT10, is also found on the surface of endothelial cells [16, 17], possessing the ability as a receptor to bind other molecules. It has reported that KRT1 associated with urokinase plasminogen activator receptor as a multiprotein receptor for high-molecular-weight kininogen, prekallikrein and 2-chain urokinase plasminogen[16]. KRT1 was also reported to serve as a receptor for fatty acid-binding protein 4[18]. Recent study showed that the SNPs of KRT1 interval is associated with the migration rates of keratinocyte when the epidermal cell undergoing wounding healing stages[19]. KRT1 plays a crucial role in maintenance of skin integrity and involvement in inflammatiory response [20]. Numerous mutations in *KRT1* have been reported [21] and most of the 62 known single nucleotide polymorphisms (SNPs) in the coding region of *KRT1* are associated with skin diseases [22]. As pathologies of SLE and SSc often involved the skin [23, 24], we hypothesized the polymorphism of KRT1 might associate with SLE and SSc as genetic factors.

KRT1 genotyping were previously analyzed in three normal populations by Han and colleagues using Sanger sequencing-based typing (SBT). Of known SNPs, two polymorphic sites in exon 9 of *KRT1* (rs14024, SNP, A/G and rs267607656 (cds-indel, -/GGCTCCGGAGGTAGCAGCTAC) were varied among different population. Other *KRT1* variants were either dominant (> 99.5%) or rare (< 0.5%) in allele frequencies. Several common haplotypes identified with family analysis mainly consist of such two polymorphic sites [25]. We focused on two polymorphic sites in exon 9 of *KRT1* (rs14024 and rs267607656) and developed a novel method based on PCR using the sequence specific primers (PCR-SSP) for KRT1 genotyping. The KRT1 genotyping was performed on the cohorts of SLE and SSc patients and healthy controls. Here, we present data to show the evidence of association between two major autoimmune diseases (SLE and SSc) and *KRT1* genotypes.

## Materials and methods

### Subjects

418 South-Chinese Han (SCH) blood donors as the normal healthy controls consisted of 76(18.2%) males and 342 (81.8%) females with age of 35.6 ± 9.3 years (Mean ± SD). 164 SLE cases (males: 6.1%, females: 93.9%; age: 30.3±8.8) and 99 SSc cases (males: 35.4%, females: 64.6%; age: 50.1±10.3) were recruited from the Xiangya Hospital from January 1, 2015 to December 31, 2015. All SLE patients defined as the cases were diagnosed using the revised criteria for the classification of SLE from the American College of Rheumatology/European League [26, 27]. 99 consecutive SSc cases were selected as SSc group based on the classification criteria for SSc of American College of Rheumatology /European League [26, 27]. Clinical information for all cases was collected from the affected individuals through a comprehensive clinical checkup by physician specialists and was tabulated in the database. Clinical experimental data were achieved from the clinic laboratory reports. Additional demographic information was gathered from cases and controls through a structured questionnaire survey. All individuals in the control group were clinically assessed to be without SLE, SSc, other autoimmune diseases, other skin diseases, or any family members (including first, second, and third degree relatives) who have the history of autoimmune and skin disorders. The study was approved by the Institutional Ethical Committee of Xiangya Hospital (201403157) and was conducted according to the Declaration of Helsinki principles. All participants provided written informed consent.

### Genotyping

Peripheral blood mononuclear cells (PBMCs) were isolated from venous blood samples with anti-coagulated acid citrate and dextrose (ACD). DNA specimens were extracted from PBMCs using QIAamp DNA Blood Mini Kits (QIAGEN China (Shanghai) Co., Ltd.). PCR-SSP was used to identify the single-nucleotide polymorphism (SNP, rs14024, A/G) and the indel polymorphism (cds-indel, rs267607656, -/GGCTCCGGAGGTAGCAGCTAC) in exon 9 of the KRT1 gene. Three sets of primer pairs were used as following: rs14024-A: 5’-CTGTGAGCACAAGCCACACC-3’ (forward) and: 5’-GGAATAAGTGGTAGAAACAAACT-3’ (reverse); rs14024-G: 5’-CTGTGAGCACAAGCCACACC-3’ (forward) and 5’-GGAATAAGTGGTAGAAACAAACC-3’ (reverse); and rs267607656: 5’-CTGTGAGCACAAGCCACACC-3’ and 5’-TCCGGAGCCGTAGCTGCCATG-3’. The primers for GAPDH, 5’-CCCTTTGAGTTTGATGATGC-3’ and 5’-GGAAGATGGTGATGGGATTT-3’ were used as internal positive control.

Each PCR reaction with 20 ul of total volume contained 1 x PCR buffer, 0.2 mM each of four deoxy-nucleotides, 1 mM each of forward and corresponding reverse primers, 0.3 units of high fidelity Taq polymerase (Roche Diagnostics, Indianapolis, IN, USA), and 50 ng genomic DNA. The PCR reaction was performed using the following thermal cycling conditions: 1 cycle at 95°C for 2 min, 30 cycles of 95°C for 30 s, 65°C for 60 s, and 72°C for 40 s respectively, and finally incubated for 10 min at 72°C. The fragments of the PCR products were visualized in 2.0% agarose gel under UV light and recorded by photograph. The SNP polymorphism at rs14042 was detected with presence or absence of PCR product, and the indel polymorphism (rs267607656, -/GGCTCCGGAGGTAGCAGCTAC) with or without 21-nucleotide deletion in exon 9 of the KRT1 gene was demonstrated with the different product size(s) in the electrophoresed gels.

### Haplotype determination

The haplotypes formed by two polymorphic sites of rs14024 and rs267607656 at KRT1 gene were estimated by Haploview software in version 4.2 (Broad Institute of MIT and Harvard, Cambridge, Barrett et al., 2005). The name of haplotypes with two letters (G-L, A-L or A-Del) represent the combination of two polymorphic sites of KRT1 gene.

### Statistical analysis

Allele and genotype frequencies of SNP and indel polymorphism were calculated. Hardy-Weinberg Equilibrium (HWE) testing was performed using Arlequin version 3.11[28]. Statistical significance between patients and controls was determined using a standard chi-square test (SPSS 18.0 software, IBM Corporation, NY, United States), or 2-df chi-square test (3 X 2). SNP data were edited into Haploview format [29] and the linkage disequilibrium (LD) and haplotype map were obtained using Haploview version 4.2. The statistical power (1-β) was calculated using PS Software (Power and Sample Size Calculation) [30]. A *p* value less than 0.05 was considered significant.

## Results

### Validation of KRT1 genotyping assay

**Fig 1:**
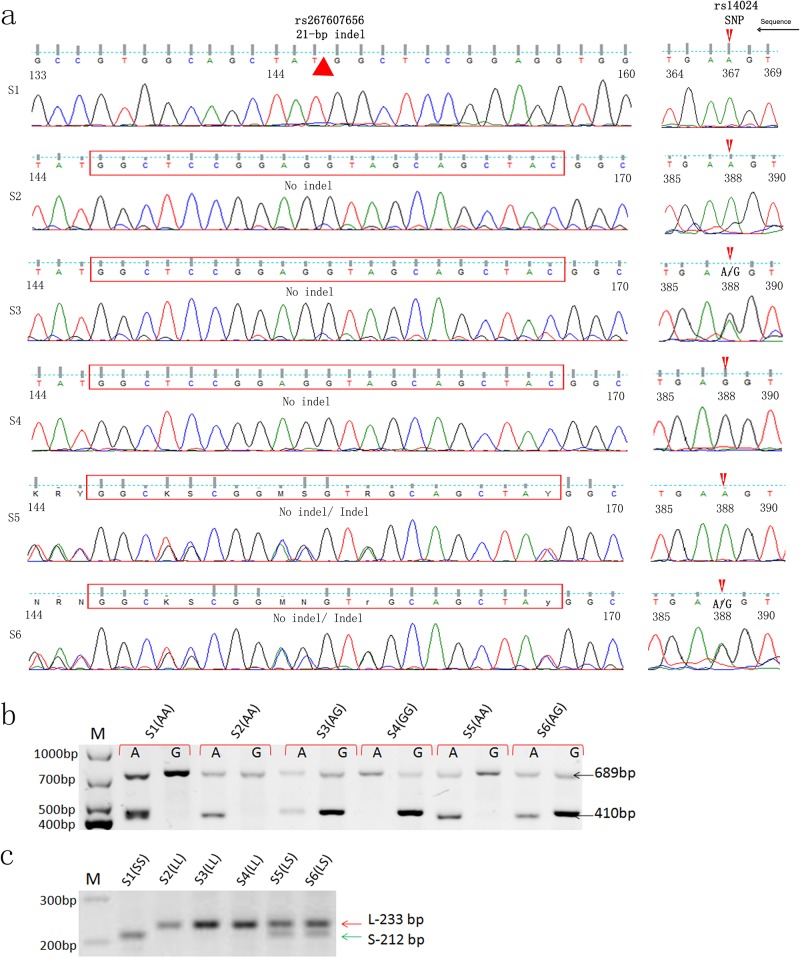
Validation of PCR-SSP genotyping assay. 6 reference DNA samples identified with Sanger sequence-based typing (SBT) are utilized to validate the new method of PCR-SSP typing. (a): PCR products with KRT1 exon 9 DNA sequences were sequenced with forward and reverse sequencing primers. The 21-bp deletion and non-deletion in 6 samples are labeled in the left profiles of reverse-primer sequencing. The SNPs rs14024 with A, G or both (A/G) at position 388 in exon 9 of KRT1 gene are given in the right side. The same samples were used to type by PCR-SSP. (b): A 689 bp band, amplified by GAPDH primers serves as a positive control. Typing of SNP with either A, G or both at rs 14024 is read based on the presence of a 410-bp band in PCR products in different primer pairs. (c): The indel polymorphism in rs267607656 of KRT1 is detected by the sizes of PCR gels with larger bands (L, no deletion) and/or small bands (S, with deletion). The relatively small size (S) detected is stand for the existing of a 21-bp deletion in KRT1 gene.

The genotypes of KRT1 were determined based on two polymorphic sites within exon 9. The genotyping of 6 reference samples with known-genotypes, which were typed using Sanger Sequencing of PCR products, were given in Fig 1. SNP rs14024 with A or G or both at position 388 of KRT1 exon 9 and indel genotypes at rs267607656 were determined with sequencing profiles (Fig 1a). The reference samples were used to validate this novel assay. A 698 bp of GAPDH gene fragment amplified in each PCR test was used as the internal positive control. Specific PCR product with or without a 410 bp size in gel represented either A or G, or both in the SNP, rs14024 (Fig 1b). 21-nuecletide deletion at rs267607656 of KRT1 gene was identified based on the size difference of PCR product running in 2% gel as Fig 1c. These typing results of 6 reference samples completely matched the results of genotypes with Sanger sequencing-based typing (SBT). The approach of PCR-SSP for KRT1 genotyping was validated.

### The frequencies of KRT1 genotypes

**Fig 2:**
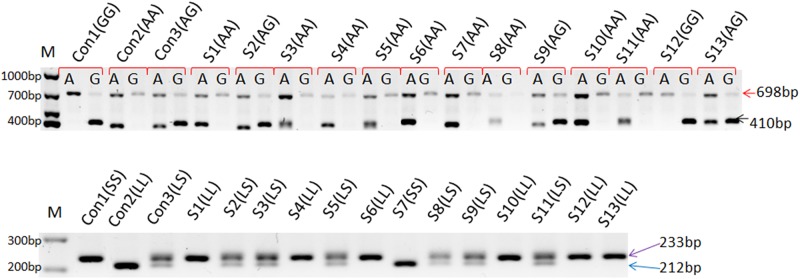
The genotyping results of samples by PCR-SSP. (a): Typing for SNP rs14024 is based on the two PCR reactions using specific sequence primers, SNP with A or G, or both were designed with a size band of 410 bp in PCR; a 689 bp band serves as the internal positive control. (b): The 21bp nucleotide deletion was detected with a small band (S) and wild type of rs267607656 is demonstrated with a large band (L) in PRC products. The PCR results of three control samples (con1, con2 and con3) and object samples (S1, S2, S3 et al.) are given, following by the read of typing results in parenthesis.

The genotyping was assigned by the pattern of PCR reactions as showed in Fig 2. Allele and genotype frequencies of each SNP and the indel polymorphism in healthy Chinese Southern Han (CSH), SLE and SSc patients’ population were given in Tables 1 and 2. All were found to be in Hardy-Weinberg Equilibrium (p = 0.08~0.95).

**Table 1:** The allele frequencies of SNP rs14024 and indel rs267607656 in different populations.

SNP	Allele	Control(2n = 836)		SLE(2n = 328)				SSc(2n = 198)			
		No.	%	No.	%	OR(95%CI)	P	No.	%	OR(95%CI)	P
rs14024	A	463	55.38	139	42.38	-	-	79	39.90	-	-
	G	373	44.62	189	57.62	1.69(1.30–2.12)	6.48×10^−5^^a^	119	60.10	1.87(1.36–2.56)	8.75×10^−5^^a^
rs267607656	L	595	71.37	259	78.96	-	-	165	83.33	--	-
	S	241	28.63	69	21.04	0.66(0.49–0.89)	0.007^a^	33	16.67	0.49(0.33–0.74)	4.89×10^−4^^a^

^a^P-value<0.05.

Allele at SNP rs14024 is either A or G. As allele at indel rs267607656, S (short) is representative to have a 21-bp deletion, and L(long) without deletion.

**Table 2:** The genotype frequencies of SNP rs14024 and indel rs267607656 in different populations.

SNP	genotype	Control(n = 418)		SLE(n = 164)				SSc(n = 99)			
		No.	%	No.	%	OR(95%CI)	P	No.	%	OR(95%CI)	P
rs14024	A/A	149	35.65	29	17.68	-	-	20	20.20	-	-
	A/G	165	39.47	81	49.39	1.50(1.04–2.15)	0.03^a^	39	39.39	0.99(0.63–1.56)	0.99
	G/G	104	24.88	54	32.93	1.48(1.0–2.19)	0.05^a^	40	40.41	2.04(1.29–3.24)	0.002^a^
rs267607656	L/L	208	49.76	103	62.80	-	-	70	70.71	-	-
	L/S	179	42.82	53	32.32	0.64(0.44–0.93)	0.02^a^	25	25.25	0.45(0.28–0.74)	0.001^a^
	S/S	31	7.42	8	4.88	0.64(0.29–1.42)	0.27	4	4.04	0.50(0.17–1.46)	0.2

^a^P-value<0.05.

Allele at SNP rs14024 is either A or G. As allele at indel rs267607656, S (short) is representative to have a 21-bp deletion, and L(long) without deletion.

The univariate analysis showed the frequencies of allele rs14024-G of KRT1 were significantly increased in the SLE patients (57.62% vs 44.62%, OR: 1.69, 95%CI: 1.30–2.12, p = 6.48×10^−5^) and SSc (60.10% vs. 44.62%, OR: 1.87, 95%CI: 1.36–2.56, p = 8.75×10^−5^) against the normal controls (Table 1), while the frequencies of the deletion allele (S) at indel rs267607656 were significantly lower in SLE and SSc patients than that in normal healthy population (OR: 0.66, 95%CI: 0.49–0.89, p = 0.007 and OR: 0.49, 95%CI: 0.33–0.74, p = 4.89×10^−4^, respectively, Table 1). The sample size used for these comparisons provided a power of about 0.98, 0.98, 0.76, and 0.96 to detect ORs of 1.69, 1.87, 0.66, and 0.49 respectively, at a type 1 error rate *α* of 0.05.

Among three genotype categories in controls and cases with SLE or SSc, the significance was also found between control and SLE group with genotypes of SNP rs14024 (A/G (OR: 1.50, 95%CI: 1.04–2.15, p = 0.03), G/G (OR: 1.48, 95%CI: 1.02–2.19, p = 0.05), Table 2). The same phenomenon is found between control and SSc group with genotype at SNP rs14024 (G/G (OR: 2.04, 95%CI: 1.29–3.24, p = 0.002), Table 2). The genotype frequencies of Indel rs267607656 (L/S) were also significantly difference between SLE group and control (OR: 0.64, 95%CI: 0.44–0.93, p = 0.02, Table 2), and SSc group and control group (OR: 0.45, 95%CI: 0.28–0.74, p = 0.001, Table 2). The sample size used for these comparisons provided a power of about 0.58, 0.48, 0.85, 0.63 and 0.92 to detect ORs of 1.50, 1.48, 2.04, 0.64 and 0.45 respectively, at a type 1 error rate α of 0.05.Next, we did further analysis on the haplotype to elucidate the effect of the two SNPs linkage.

#### KRT1 haplotype analysis

The haplotype combining two polymorphic sites tested was estimated with conventional estimation and maximization (EM) algorithm (significance level setup to 0.05) among three populations. Three of four possible haplotypes were found. As shown in Table 3, wild type (WT, A-L), combining allele-A at rs14024 and no deletion at rs267607656, deletion mutant (Del, A-S) with allele-A at rs14024 linking to 21-neucletide deletion at rs267607656, and mutant (MU, G-L) with G substitution at rs14024 without deletion mutant, were given in Table 3. None of the haplotype MD (G-S) was observed among three groups, while this haplotype MD combining a mutant “G” and 21-nueculetide deletion was supposed to be found in populations. The linkage disequilibrium (LD) of two sites with two alleles was evaluated in each group. Since three of four possible haplotypes were observed, the results showed that two polymorphic sites were in complete linkage disequilibrium in all three groups (D’ = 1 and r^2^ = 1), mainly due to the absence of haplotype MD (G-S).

**Table 3:** The frequencies of haplotype of KRT1 gene in SLE, SSc and control group.

Haplotype	Control(2n = 836)	SLE(2n = 328)				SSc(2n = 198)			
	No.(%)	No.(%)	OR(95%CI)	P^a^	1-β^b^	No.(%)	OR(95%CI)	P	1-β^b^
WT(A-L)	222(26.56)	70(21.34)	0.75(0.55–1.02)	0.07	0.47	46(23.23)	0.84(0.52–1.20)	0.34	0.15
MU(G-L)	373(44.62)	189(57.62)	1.69(1.30–2.19)	6.48×10^−5^^a^	0.98	119(60.10)	1.87(1.36–2.56)	8.75×10^−5^^a^	0.98
DEL(A-S)	241(28.83)	69(21.04)	0.66(0.49–0.89)	0.007^a^	0.76	33(16.67)	0.49(0.33–0.74)	4.89×10^−4^^a^	0.96

^a^P-value<0.05.

1-β^b^: statistic power was calculated at a type 1 error rate α of 0.05.

Haplotype MD (G-S) is not found in all samples tested.

### Haplotype of KRT1 and disease association

Considering two polymorphic sites as a haplotype of KRT1, the haplotypic frequencies of wild type (WT, A-L) were not different in SLE and SSc groups against control (p = 0.07 and 0.34, respectively, Table 3). The frequencies of the mutant haplotype (KRT1-MU, G-L) were significantly higher in SLE (57.62% vs. 44.62%, OR: 1.69, 95%CI: 1.30–2.19, p = 6.48×10^−5^) and SSc (60.1% vs. 44.62%, OR: 1.87, 95%CI: 1.36–2.56, p = 8.75×10^−5^) than that in the control (Table 3). However, the frequency of the deletion haplotype (KRT1-Del, A-S) was significantly lower in the SSc (16.67% vs. 28.83%, OR: 0.49, 95%CI: 0.33–0.74, p = 4.89×10^−4^, Table 3) than that in the control group. Since the frequency of haplotype Del was significantly lower in the SSc group, the deletion of 21 nucleotides at rs267607656 might associate with resistance to, or protection from SSc.

### Haplogenotype of KRT1 and disease association

Hypothesizing that the haplotypes of KRT1-Del (low risk) and KRT1-MU (high risk) haplotypes have opposite effects to associate with SLE and SSc as above, the patients were further classified into three sub-groups according to the individuals having Del and no MU genotype (Del+/MU-, low risk alone), having no Del but with MU genotype (Del-/MU+, high risk alone), having both haplotypes (Del+/MU+, opposite risk together) and none of them (Del-/MU-) for a deep analysis (Table 4). The haplogenotype of Del-/MU+ were associated with a high risk to SLE (OR: 1.87, 95%CI: 1.30–2.69, p = 0.001) and SSc (OR: 2.29, 95%CI: 1.47–3.60, p = 2.32×10^−4^) observed as expected. Interestingly, the haplogenotype Del+/MU- were associated with the resistance to both SLE (OR: 0.35, 95%CI: 0.21–0.60, p = 6.24×10^−5^) and SSc (OR: 0.34, 95%CI: 0.17–0.66, p = 0.001) as predicted (Table 4). However, if two opposite haplotypes (Del+/MU+) present together, there was no significance found in subgroups with either SLE (OR:1.139, 95%CI: 0.750–1.730, p = 0.541) or SSc (OR:0.735, 95%CI: 0.421–1.286, p = 0.280)(Table 4). These results supported our hypothesis.

**Table 4:** The distribution of different haplo-genotypes in SLE, SSc and control group.

genotype	Control (n = 418)	SLE(n = 164)				SSc(n = 99)			
	No.(%)	No.	OR(95% CI)	P^a^	1-β^b^	No.	OR(95% CI)	P^a^	1-β^b^
Del-/MU+	172(41.15)	93(56.71)	1.87(1.30–2.69)	0.001^a^	0.88	61(61.62)	2.29(1.47–3.60)	2.32×10^−4^^a^	0.94
Del+/MU-	113(27.03)	19(11.59)	0.35(0.21–0.60)	6.24×10^−5^^a^	0.97	11(11.11)	0.34(0.17–0.66)	0.001^a^	0.93
Del+/MU+	97(23.21)	42(25.61)	1.14(0.75–1.73)	0.541	0.07	18(18.18)	0.74(0.42–1.29)	0.280	0.27
Del-/MU-	36(8.61)	10(6.1)	0.62(0.26–1.42)	0.312	0.30	9(9.09)	1.11(0.49–2.28)	0.897	0.05

^a^P-value<0.05.

1-β^b^: statistic power was calculated at a type 1 error rate α of 0.05.

## Discussion

After reviewing the SNPs of KRT1 and their allelic frequencies in populations, two polymorphic sites in exon 9 of KRT1 are highlighted for the analysis in our investigation. SNP rs14024 encodes a nonsynonymous amino acid change from a lysine to arginine in exon 9 because of the codon replacing from AAG to AGG). The coded amino acid is positioned in the carboxy tail region of KRT1 molecule and this location is away from the highly conserved α-helical rod domains in the center of the KRT1 protein [19]. Indel polymorphism at rs267607656 with two size variants of human keratin 1 protein chain described by Korge [31] is characterized by a deletion of 7 amino acids in a repeatable sequence of the transmembrane subdomain of KRT1. This deletion corresponds to loss of an entire glycine loop of seven amino acids. The PCR-based KRT1 allele typing showed the different patterns with three PCR products (Fig 1). Our data also indicate that both polymorphic sites frequently varied in our local population (Table 1).

Our data demonstrated that the allele of rs14024-G was more frequent in patients with SLE and SSc than that in the local normal population (p = 6.48×10^−5^ and p = 8.75×10^−5^, Table 1). And the analysis of genotypes of the SNP rs14024 showed that the G/G was associated with SSc (OR: 2.04, 95%CI: 1.29–3.24, p = 0.002). The frequencies of allele with 21bp deletion (S) in KRT1 were lower in SSc patients than that in the local normal population. Moreover, the ratio of deletion-homozygous (S/S) at rs267607656 was also decreased in SSc patient groups comparing to the control (Table 2). Since it was difficult to identify the association of two alleles with the opposite effects, further analysis was performed on tran-cis for haplotypes of KRT1 gene.

Possible four haplotypes were supposed to be observed if two sites with two alleles in each. Our results showed that the haplotype MD (G-S) (Table 3) was not found in any of the samples tested. The same results have been reported by Dr. Stastny’s group [25]. It might be due to the short distance between two sites at KRT1 gene, as the crossover might not happen. In other possibility this kind of haplotype might be lethal. Our data indicated that mutation allele rs14024-G was high risk to associate with SLE (p = 6.48×10^−5^) and SSc (p = 8.75×10^−5^) (Table 1), but the frequency of deletion (S) at rs267607656 showed the resistant trend to SSc (OR: 0.49, 95%CI: 0.33–0.74, p = 4.89×10^−4^, Table 1). These results generated our hypothesis: haplotype MU (G-L) is susceptible to SLE and SSc and haplotype Del (A-S) is resistant to them. To analyze the data with this idea, we clearly showed that the frequency of haplogenotype of Del-/MU+ was significantly higher in SLE (p = 0.001) and SSc (p = 2.32×10^−4^) than that in control (Table 4). In contrast, the haplogenotype with a 21-base deletion but not with G mutation (Del+/Mu-) was significantly lower in SLE and SSc than that in control (p = 6.24×10^−5^, p = 0.001, respectively, Table 4). As expected, no difference was observed between the control and the patients with Del+/MU+ haplogenotype, as having an opposite effect with two haplotypes. This detail analysis avoided the neutralization or counteraction with opposite effect on tran-cis polymorphic sites.

Based on the GWAS study, Protein C-ets-1, DNA-binding protein Ikaros, Ras guanyl-releasing protein 3, Solute carrier family 15 member 4, TNF-α-induced protein 3-interacting protein 1 (TNIP1), 7q11.23, 10q11.22, 11q23.3 and 16p11.2 were identified associating with SLE susceptibility in a Chinese Han population [5]. A slice of genetic loci were also confirmed associating with SSc susceptibility by GWAS, such as CD247 [32], TNIP1, Psoriasis susceptibility 1 candidate gene 1 protein, and Rho-related GTP-binding protein RhoB[33]. Among them, several variants were reported involving in the proliferation of keratinocyte [34, 35]. As a major protein produced by keratinocyte, keratins were reported participated in the progress of inflammation [20]. Given the skin inflammation was a common symptom of SLE and SSc, we genotyped the KRT1 and explored their association.

Up to now, GWAS have identified more than 80 SLE susceptibility loci [36] and 40 SSc risk genetic regions [37]. Nevertheless, the causal mutations responsible for the diseases remain elusive, it is necessary to conduct a more detailed investigation of the individual gene. Among the founded susceptibility loci, variants on Interferon-induced helicase C domain-containing protein 1 (IFIH1) and Complement C1r subcomponent (C1R) attracted our attention. IFIH1 is a double-strand RNA receptor and play a vital role in innate immune response during virus infection by inducing the expression of interferon.[38] Polymorphisms in IFIH1 were also shown association with Aicardi-Goutières syndrome [39], type 1 diabetes mellitus[40] and psoriasis[41]. It was reported that IFIH1 mutations resulted in the aberrant induction of type I interferon, which was an etiology of Aicardi-Goutières syndrome [42]. Previous studies had shown that the severity of SLE was associated with the level of type I interferon[43, 44],consistent with other researcher’s finding that the variants of IFIH1 were a genetic risk for SLE[45, 46]. The levels of serum complement are reported to link to the pathogenesis of SLE [47] and Demirkaya E et al. shown that the variant in C1R resulting in a dysfunction complete C1r lead to the occurrence of the SLE [48]. The study of a single gene is conductive to explore more detail information about pathogenesis.

Our data showed that the association between two KRT1 polymorphic sites and effects of autoimmune diseases SLE and SSc. More work need to be done to elucidate the genetic diversity of KRT1 and the association with immune diseases. Some of such research work had been done and were reported by others. Pant and colleagues [49] found the KRT1 has extreme allele-specific expression differences in human white blood cells. This differential allelic expression of KRT1 is predominantly controlled by cis-regulatory polymorphism(s) in strong linkage disequilibrium with the gene.

Recently study by Roth et al. revealed a KRT1-mediated gene expression signature similar to atopic eczema and psoriasis, suggesting a functional link between KRT1 and human inflammatory skin diseases [20], and Wallace [50] reported that deletion of K1/K10 affects desmosomal structure and nuclear integrity. It is interesting to understand whether these specific alleles of KRT1 affect the structure of the encoded protein and hence its functions. This information is very useful for us to explore that KRT1 gene variety involves in the pathogenesis of SLE, SSc and other autoimmune diseases. However, expanding our study in a large cohort is needed to confirm our finding.

The data generated from our finding and others’ investigation might lead us to measure the amount of the expression of different haplotype of KRT1 in the further study. In our previously study [51], we had detected the antibodies against KRT1 among patients with autoimmune diseases using the antigens encoded by three common KRT1 alleles mentioned in this paper. The distinct epitopes encoded by the specific KRT1 alleles are identified to serve as targets for the antibodies against KRT1 molecules in patients. Therefore, typing for KRT1 genotypes could improve the diagnosis for SLE and SSc, and helpful to identify high risk individuals with potential genetic background. Moreover, it might be benefit to development of genetic therapy for such autoimmune diseases.
